# Capecitabine and oxaliplatin combined with bevacizumab are feasible for treating selected Japanese patients at least 75 years of age with metastatic colorectal cancer

**DOI:** 10.1186/s12885-015-1712-0

**Published:** 2015-10-24

**Authors:** Yoshinori Munemoto, Mitsuro Kanda, Keiichiro Ishibashi, Taishi Hata, Michiya Kobayashi, Junichi Hasegawa, Mutsumi Fukunaga, Akinori Takagane, Toshio Otsuji, Yasuhiro Miyake, Michitaka Nagase, Junichi Sakamoto, Masaki Matsuoka, Koji Oba, Hideyuki Mishima

**Affiliations:** 1 Department of Surgery, Fukuiken Saiseikai Hospital, Fukui, Japan; 2 Department of Gastroenterological Surgery (Surgery II), Nagoya University Graduate School of Medicine, 65 Tsurumai-cho, Showa-ku, Nagoya, 466-8550 Japan; 3 Department of Digestive Tract and General Surgery, Saitama Medical Center, Saitama Medical University, Kawagoe, Japan; 4 Department of Gastroenterological Surgery, Osaka University Graduate School of Medicine, Osaka, Japan; 5 Department of Human Health and Medical Sciences, Kochi Medical School, Kohasu, Japan; 6 Department of Surgery, Osaka Rosai Hospital, Sakai, Japan; 7 Department of Surgery, Hyogo Prefectural Nishinomiya Hospital, Nishinomiya, Japan; 8 Surgical Division, Hakodate Goryoukaku Hospital, Hakodate, Japan; 9 Department of Internal Medicine, Dongo Hospital, Yamatotakada, Nara Japan; 10 Department of Surgery, Minoh City Hospital Gastrointestinal Research Center, Minoh, Osaka Japan; 11 Department of Surgical Oncology, Gifu University Graduate School of Medicine, Gifu, Japan; 12 Director, Tokai Central Hospital, Gifu, Japan; 13 Matsuoka Clinic, Kitakatsuragi, Nara Japan; 14 Department of Biostatistics, School of Public Health, Tokyo University Graduate School of Medicine, Tokyo, Japan; 15 Interfaculty Initiative in Information Studies, Tokyo University, Tokyo, Japan; 16 Unit of Cancer Center, Aichi Medical University, Nagakute, Japan

**Keywords:** Colorectal cancer, Elderly, Bevacizumab, XELOX

## Abstract

**Background:**

Although number of elderly patients with metastatic colorectal cancer (mCRC) is rapidly increasing, this population is often underrepresented in clinical trials. Recently, a phase II trial demonstrated that capecitabine and oxaliplatin (XELOX) combined with bevacizumab XELOX plus bevacizumab was effective and well tolerated by elderly patients with mCRC who reside in Western countries. The aim of this study was to evaluate the safety and efficacy of XELOX plus bevacizumab for Japanese patients aged ≥75 years with mCRC.

**Methods:**

This prospective, open-label phase II trial recruited patients aged ≥75 years with previously untreated mCRC between March 2010 and January 2012. Treatment consisted of 7.5 mg/kg of intravenous bevacizumab and 130 mg/m^2^ of oxaliplatin on day 1 of each cycle combined with 2000 mg/m^2^ of oral capecitabine per day on days 1–14 of each cycle. Treatment was repeated every 3 weeks until disease progression or termination of the study. The primary endpoint was progression-free survival; the secondary endpoints were toxicity, overall response rate, time-to-treatment failure, and overall survival.

**Results:**

Thirty-six patients (male 58 %; median age 78 years; colon cancer 67 %) met all eligibility criteria and received at least one course of the planned treatment. The median time-to-treatment failure was 7.0 months. Twelve patients (33.3 %) experienced adverse effects (AEs) ≥ grade 3 and frequent AEs ≥ grade 3, including neutropenia (22.2 %) and neuropathy (13.9 %). Hypertension was the most frequent AE ≥ grade 3 associated with bevacizumab (11.1 %). Low baseline creatinine clearance associated significantly with the incidence of AEs ≥ grade 3. Response and disease control rates were 55.6 and 91.7 %, respectively. Median progression-free and overall survival times were 11.7 months (95 % confidence interval, 8.0–13.4 months) and 22.9 months, respectively.

**Conclusion:**

XELOX combined with bevacizumab was well tolerated by selected Japanese patients aged ≥75 years with mCRC patients, and controlled clinical trials are now required to determine the survival benefit.

**Electronic supplementary material:**

The online version of this article (doi:10.1186/s12885-015-1712-0) contains supplementary material, which is available to authorized users.

## Background

Colorectal cancer ranks worldwide as the third and fourth most common cancer in women and men, respectively, and the median survival of patients with metastatic colorectal cancer (mCRC) treated with best supportive care is approximately 6 months [1–3]. Treatment outcomes are improved considerably by newly developed chemotherapeutic agents and regimens. For example, treatment using 5-fluorouracil (5-FU) plus irinotecan, oxaliplatin, or both combined with targeted agents extends median overall survival (OS) to approximately 30 months [4, 5]. Current guidelines recommend that first-line treatment for patients with mCRC should include doublet chemotherapy plus a targeted agent, if tolerated [6].

The prodrug capecitabine is activated by a unique mechanism that exploits the high activity of thymidine phosphorylase in malignant tissue that generates 5-FU preferentially in tumor tissue [7]. Capecitabine undergoes a three-step enzymatic conversion, and the final stage is catalyzed by thymidine phosphorylase, which is significantly more active in tumor tissue compared with healthy tissue [7, 8]. Oral delivery of capecitabine simplifies chemotherapy and provides convenient outpatient therapy, because it avoids the complications and discomfort associated with intravenous administration and permits prompt discontinuation of treatment when toxicity occurs [9].

Combining capecitabine with oxaliplatin (XELOX) is advantageous for the reasons as follows: synergistic effects, no overlapping toxicities, easy to administer, and outpatient management [10–13]. Randomized phase III trials demonstrate that outcomes using first-line XELOX are comparable with those achieved using continuous infusion of 5-FU and folinic acid combined with oxaliplatin (FOLFOX) [14, 15]. Moreover, combined with bevacizumab, a recombinant humanized version of a mouse monoclonal antibody against human vascular endothelial growth factor, XELOX achieves significantly improved progression-free survival (PFS) compared with chemotherapy alone [16–18].

The average age of the population is steadily increasing in many developed countries, particularly because of improvements in public health, nutrition, disease prevention, early detection, and continued progress in medical research [19]. The increase in patients’ ages presents the medical community with new challenges. For example, more than 30 % of patients with newly diagnosed CRC are aged at least 75 years [20]. Since the progressive reduction of functional reserve that occurs in various organs with ageing might increase the susceptibility of the elderly to adverse effects, clinical trials for elderly patients with mCRC have been conducted and tolerability of UFT/leucovorin, XELOX, capecitabine plus bevacizumab and S-1 plus bevacizumab were evaluated [21–26]. Yet, the safety and efficacy of XELOX plus bevacizumab for elderly patients remains to be determined because earlier large clinical trials limited eligibility to individuals <70 or 75 years owing to frail health [16, 17, 27]. Recently, a phase II trial (BECOX study) found that XELOX combined with bevacizumab is effective and well tolerated by patients aged ≥70 years with mCRC who reside in Spain [28]. However, insufficient evidence is available to establish the safety and benefit of XELOX plus bevacizumab for Japanese patients with mCRC included in this age group. Moreover, lack of robust evidence of the new treatment described above may subject patients of advanced age to more conservative and less effective treatments. For example, older patients are more likely to receive monotherapy instead of combination therapy that does not include agents that target specific molecules [29–31].

Therefore, the aim of the present study was to evaluate the feasibility of XELOX plus bevacizumab for selected Japanese patients with mCRC aged ≥75 years.

## Methods

### Patients and methods

A single-arm multicenter phase II trial (ASCA trial, Avastin plus XELOX Strategy for elderly patients with metastatic colorectal cancer) was planned to evaluate the safety and efficacy of XELOX plus bevacizumab for patients with mCRC ≥75 years of age [32]. The scientific and ethical validity of the study protocol was reviewed and approved by an internal review board of each participating facility (the Institutional Review Board at Osaka National Hospital, Osaka City General hospital, Osaka Rosai Hospital, Kitakyushu General Hospital, Kinki University, Kochi University, Fukui-ken Saiseikai Hospital, Saitama Medical Center, Jichi Medical University, Izumisano Municipal Hospital, Sakai City Hospital, Toyonaka Municipal Hospital, Dongo Hospital, Nara Social Insurance Hospital, Hakodate Goryoukaku Hospital, Fukuiken Saiseikai Hospital, Minoh City Hospital and Mimihara General Hopital). Written informed consent was obtained from all patients before enrollment. This study was conducted in accordance with the Declaration of Helsinki (2008) and registered with the University Hospital Medical Information Network (UMIN) Clinical Trial Registry as UMIN000003500 (http://www.umin.ac.jp/ctr/index.htm).

Patients from 18 institutes were included in this study if they met all eligibility criteria as follows: (1) written informed consent before treatment; (2) age ≥75 years when informed consent was granted; (3) Eastern Cooperative Oncology Group (ECOG) Performance Status (PS) of 0 or 1; (4) life expectancy >3 months; (5) histologically confirmed colorectal adenocarcinoma; (6) measurable disease consistent with the Response Evaluation Criteria in Solid Tumors (RECIST) version 1.1; (7) no prior chemotherapy (adjuvant chemotherapy included fluorouracil and/or oxaliplatin was allowed, but the last course of adjuvant chemotherapy must have concluded more than six months prior to colorectal cancer recurrence); (8) adequate function of vital organs, including liver and kidney (total bilirubin ≤1.5-times the institutional upper normal limit, aspartate aminotransferase and alanine aminotransferase ≤2.5-times the institutional upper normal limit, and serum creatinine ≤ institutional upper normal limit or creatinine clearance (CCr, calculated using the Cockcroft–Gault formula) ≥50 ml/min); adequate bone marrow function (leucocyte count ≥3000/mm^3^, neutrophil count ≥1500/mm^3^, platelet count ≥100,000/mm^3^, and hemoglobin ≥9.0 g/dl).

Key exclusion criteria included uncontrolled pleural effusion or ascites, brain metastasis, presence of other active malignancies, present or past (within the past 1 year) clinically significant cerebrovascular disease or thromboembolism, surgery planned during the course of the trial, anticoagulant treatment, coagulation disorder, nephropathy requiring medication or transfusion, uncontrolled hypertension or diabetes mellitus, uncontrolled diarrhea, history of bevacizumab treatment, and inability to take drugs orally [32].

### Treatment

Treatment consisted of intravenous administration of 7.5 mg/kg of bevacizumab and 130 mg/m^2^ of oxaliplatin on day 1 of each cycle combined with 2000 mg/m^2^ oral capecitabine per day on days 1–14 of each cycle [32]. The end of the protocol treatment period was not prescribed. Treatment was repeated every 3 weeks until disease progression or termination of the study. The study protocol had no provisions regarding the second-line treatment. When patients exhibited adverse effects (AEs), the dose of each drug was reduced as specified in the study protocol that provided detailed algorithms to manage drug-specific toxicities such as oxaliplatin-related neuropathy, capecitabine-related diarrhea, hand–foot syndrome, bevacizumab-related hypertension, bleeding, and thromboembolism as well as other treatment-related toxicities. The dose reduction or stopping criteria of drugs due to adverse events is defined based on the haematological toxicity (Grade 4 neutropenia, Grade 3 febrile neutropeni a or Grade 3 or more decrease in platelets) and Grade 3 non-haematological toxicity. Dose reduction due to adverse events was performed for each drug as specified in the study protocol, which provided detailed algorithms to manage drug-specific toxicities such as oxaliplatin-related neuropathy as follows; G1, continue administration; G2/3, until recovery to G1 or less and resume oxaliplatin with the reduction dose (for the first time 100 mg/m^2^, for the second time 85 mg/m^2^); G4, discontinuation of oxaliplatin.

### Study parameters

Screening and baseline evaluations included assessing ECOG PS and conducting blood tests and physical examinations. Baseline tumor status with prospective identification of index lesions that were followed over the course of the study, was assessed using computed tomography (CT) studies of the chest, abdominal, and pelvis as well as determination of serum tumor-marker levels (carcinoembryonic antigen and carbohydrate antigen 19–9). During treatment, tumor status was assessed at the completion of each 8-week cycle. RECIST ver. 1.1 was used to evaluate responses and determine disease progression. Response rate assessment was done locally. Toxicities, graded according to the criteria of the National Cancer Institute Common Terminology for Adverse Events (version 4.0), were evaluated during the study period and for 28 days after the last dose administered during the study by conducting physical examinations and laboratory tests (hematology, chemistry and electrolytes, and urinalysis), and evaluating ECOG PS. Patients who discontinued the protocol treatment were followed every 2 months until death or loss to follow-up. Neurotoxicity was graded as follows: G1 (asymptomatic) loss of deep tendon reflexes or paresthesia, G2 (moderate symptoms) limiting instrumental activities of daily living, G3 (severe symptoms) limiting daily self-care activities; G4 (life-threatening consequences) urgent intervention indicated, and G5 (death). Patients were questioned about their use of concomitant medication and AEs. Association between the incidence of AEs ≥ G3 and baseline CCr, American Society of Anesthesiologists (ASA) score (comorbidity index), ASA Physical Status Classification System score, age, body mass index (BMI), and sex were evaluated as potential risk factors for severe AEs.

### Statistical analysis

The primary objective of the ASCA study was to determine PFS. Secondary endpoints were toxicity, overall response rate, time to treatment failure (TTF), and OS. Assuming a threshold PFS of 6.5 months and an estimated median PFS of 10.5 months, and referring to data from previous clinical trials we determined that a significance level = 95 %, an α-error = 0.05, and 32 patients were required. Estimating a loss as high as 10 % of the final subject population, 35 patients were required. The Kaplan–Meier method was used to estimate survival, and the Cox proportional hazards model was used to calculate confidence intervals (CI). PFS was defined as the interval from the time of enrolment to the date of the first documented disease progression or a patient’s death from any cause. OS was defined as the date of enrolment until the date of death from any cause. TTF was defined as the time from randomization to discontinuing treatment for any reason, including disease progression, treatment toxicity, patient preference, or death. The goodness-of-fit for AEs ≥ grade 3 was assessed by calculating the area under the curve (AUC), and optimal cutoff values were determined using the Youden index. The χ^2^ test was used to compare the difference between the values of two patient groups. A statistically significant difference was defined as *P* < 0.05.

## Results

### Patient characteristics

Thirty-seven patients treated between March 2010 and January 2012 at 18 institutes were screened and met all eligibility requirements. One patient withdrew from the study before receiving treatment. The 36 patients (male 58 %; median age 78 years; colon cancer 67 %) enrolled received at least one course of the planned treatment. Baseline patient characteristics are shown in Table 1.

**Table 1:** Baseline patient characteristics (*n* = 36)

Clinical characteristic	Number of patients (%)
Sex	
Male	21 (58.3)
Female	15 (41.7)
Age (years)	
Median (range)	78 (75–86)
ECOG performance status	
0	30 (83.3)
1	6 (16.7)
Primary sites	
Colon	24 (66.7)
Rectum	12 (33.3)
Primary tumor resection	
Performed	23 (63.9)
Not performed	13 (36.1)
Adjuvant chemotherapy	
Performed	9 (25.0)
Not performed	27 (75.0)
Appearance of metastasis	
Synchronous	19 (52.7)
Metachronous	17 (47.3)
Metastatic sites	
Liver	21 (58.3)
Lung	13 (36.1)
Lymph nodes	14 (38.9)
Peritoneum	2 (5.6)
Other	2 (5.6)
Number of metastatic sites	
1	23 (63.9)
2	11 (30.5)
3	2 (5.6)
Creatinine clearance (mL/min)	
Median (range)	60.8 (32.6–84.6)

*ECOG* Eastern Cooperative Oncology Group

### Safety and response to treatment

**Fig. 1:**
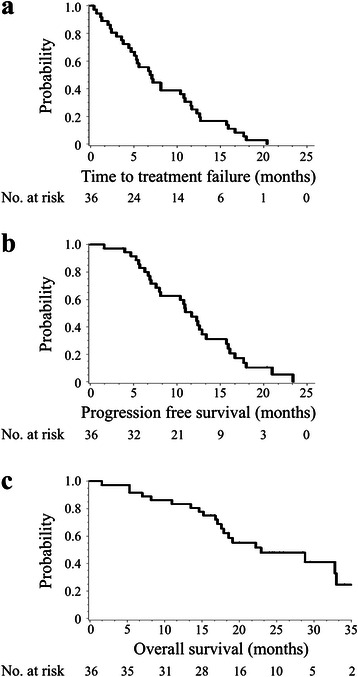
The Kaplan–Meier curves for TTF, PFS, and OS. **a** The median time to treatment failure was 7.0 months (95 % CI 4.7–10.8 months). **b** The median progression-free survival time was 11.7 months (95 % CI 8.0–13.4 months). **c** The median overall survival time was 22.9 months (95 % CI 17.6–33.0 months)

Patients were treated with a median of five cycles of XELOX plus bevacizumab (range 1–17), and the median relative dose intensities during the initial protocol (XELOX plus bevacizumab) were 86, 89, and 100 % for capecitabine, oxaliplatin, and bevacizumab, respectively. There were 14 patients who continued to receive the protocol treatment after withdrawal of oxaliplatin (capecitabine with bevacizumab for 12 and capecitabine alone for two patients). The median TTF was 7.0 months (95 % CI 4.7–10.8 months) (Fig. 1a). The reasons for discontinuing treatment were disease progression (*n =* 14), AEs (*n =* 14), withdrawal (*n =* 6), and surgery for metastases (*n =* 2). AEs that prevented continuing were as follows: neutropenia (*n* = 3), thrombotic disease (*n* = 2), anorexia (*n* = 2), ileus (*n* = 2), heart failure (*n* =1), hand–foot syndrome (*n* = 1), cerebral bleeding (*n* = 1), neuropathy (*n* = 1), and fatigue (*n* = 1).

Treatment-related toxicities are listed in Table 2. Thirty-four (94.4 %), and 12 (33.3 %) patients experienced AEs or AEs ≥ grade 3, respectively, and one treatment-related death was caused by intracranial bleeding. The latter patient was a 77-year-old woman with liver and lung metastasis without serious comorbidities who received seven courses of protocol treatment (XELOX plus bevacizumab) using the regular dose. During the eighth course, she lost consciousness, was diagnosed with intracerebral bleeding according to the results of a CT scan, and chemotherapy was discontinued. Frequent adverse events (any grade) were as follows: neuropathy (83.3 %), anemia (80.5 %), thrombocytopenia (58.3 %), hand–foot syndrome (58.3 %), and neutropenia (55.6 %). Frequent AEs ≥ grade 3 were neutropenia (22.2 %) and neuropathy (13.9 %). Bevacizumab-related AEs, proteinuria (36.1 %), and hypertension (27.8 %), were frequently observed for all grades, and the most frequent ≥ grade-3 event was hypertension (11.1 %).

**Table 2:** Treatment-related adverse events

	Grades 1/2	Grade 3	Grades 4/5	All grades (%)	≥Grade 3 (%)
Hematologic AEs					
Overall				94.4	33.3
Leucopenia	14	1	0	41.7	2.8
Neutropenia	12	7	1	55.6	22.2
Anemia	27	2	0	80.5	5.6
Thrombocytopenia	19	2	0	58.3	5.6
Non-hematologic AEs					
Elevated AST	11	0	0	30.5	0
Elevated ALT	6	0	0	16.7	0
Hyperbilirubinemia	2	0	0	5.6	0
Fatigue	7	3	0	27.8	8.3
Anorexia	9	2	0	27.8	5.6
Nausea	4	0	0	11.1	0
Vomiting	3	1	0	11.1	2.8
Diarrhea	4	2	0	16.7	5.6
Stomatitis	5	0	0	11.1	0
Hand–foot syndrome	18	3	0	58.3	8.3
Injection site reaction	3	0	0	8.3	0
Neuropathy (sensory)	25	5	0	83.3	13.9
Allergy	2	0	0	5.6	0
Bevacizumab-associated AEs					
Hypertension	6	4	0	27.8	11.1
Proteinuria	11	2	0	36.1	5.6
Thrombosis	0	1	0	2.8	2.8
Bleeding	1	1	1	8.3	5.6

*AE* adverse effect

We evaluated the association between AEs ≥ G3 and baseline patient conditions including CCr, comorbidity index, ASA Physical Status Classification System score, age, BMI, and sex. These findings identified baseline CCr as a potential predictor of AEs ≥ grade 3. The AUC value of baseline CCr = 0.69, and the optimal cutoff value for predicting AEs ≥ grade 3 = 64 ml/min (sensitivity = 0.91, specificity = 0.50 (Additional file 1: Figure S1a). Further, patients with baseline CCr <64 ml/min had a significantly higher incidence of AEs ≥ G3 compared with those with baseline CCr ≥64 ml/min (77.8 % and 22.2 %, respectively, *P* = 0.018). No association was found between evaluated factors other than CCr (comorbidity index, ASA Physical Status Classification System score, age, BMI, and sex) and incidence of AEs ≥ G3.

### Efficacy

The best radiographic response of each patient is presented in Additional file 1: Figure S1b. Responses to treatment were defined as follows: complete response (CR), partial response (PR), stable disease (SD), and progressive disease, according to the RECIST ver. 1.1 definitions. The rates for CR, PR, and SD were 2.8, 52.8, and 36.1 %, respectively, and the response and disease control rates were 55.6 and 91.7 %, respectively (Table 3). The median PFS was 11.7 months (95 % CI, 8.0–13.4 months (Fig. 1b), and the median OS was 22.9 months (95 % CI 17.6–33.0 months, Fig. 1c).

**Table 3:** Treatment profiles

Tumor response	*n* (%)
CR	1 (2.8)
PR	19 (52.8)
SD	13 (36.1)
PD	0 (0)
Not evaluated	3 (8.3)
Response rate (CR + PR)	20 (55.6)
Disease control rate (CR + PR + SD)	33 (91.7)

*CR* complete response, *PR* partial response, *SD* stable disease, *PD* progressive disease

## Discussion

Robust evidence from the TREE 1 (XELOX) and TREE 2 (XELOX plus bevacizumab) (TREE1/2) randomized clinical trials shows that XELOX combined with bevacizumab offers survival benefits to patients with mCRC [33]. Unfortunately, insufficient evidence is available to insure the safety and benefits of combined treatment with XELOX and bevacizumab for patients aged ≥75 years, that were often excluded from randomized trials, allegedly because of frail health or because they represented a minority of enrolled patients [16–18]. Feliu et al. conducted a recent phase II trial (BECOX study) in Spain and demonstrated that XELOX plus bevacizumab was effective and well tolerated by patients with mCRC aged ≥70 years [28]. Here we designed a multicenter open-label phase II trial to evaluate the safety and efficacy of XELOX plus bevacizumab for Japanese patients aged ≥75 years with mCRC. The doses of capecitabine, oxaliplatin, and bevacizumab were determined with reference to the TREE1/2 trials [33], although the median age of patients enrolled in these studies was 62 years.

In the present study, we administered a median of five cycles of treatment (XELOX plus bevacizumab) (range, 1–17). Relative dose-intensities of capecitabine, oxaliplatin, and bevacizumab during the initial protocol (XELOX plus bevacizumab) were 86, 89, and 100 %, respectively. The median TTF was 7.0 months, although TTF represents a composite endpoint influenced by factors unrelated to efficacy, because discontinuation may be due to toxicity, patient preference, or a physician's reluctance to continue therapy. These results are similar to, or somewhat better compared with those of the TREE1/2 trials as well as those of the BECOX study [28, 33], despite the older patients studied here. The results of the present study and relevant clinical trials for mCRC were summarized in Table 4. Because therapeutic regimens with or without bevacizumab do not necessarily affect relative dose intensities of capecitabine and oxaliplatin, our results are comparable with the results of trials involving younger patients indicating that XELOX plus bevacizumab is well tolerated by patients aged ≥75 years with mCRC.

**Table 4:** Summary of our results and relevant clinical trials for mCRC

Study/first author	Phase	Treatment	Country	n	ECOG PS^a^	Age (median)	RR	PFS (month)	OS (month)	Neuropathy (G3/4)	Ref
XELOX											
Cassidy J	2	XELOX	6 European, Canada	96	0–1	34–79 (64)	55 %	7.7	19.5	17 %	12
TREE-1 Hochster HS	2	mFOLFOX6 vs FOL vs XELOX	United States	150	0–1	31–84 (62)	41 % vs 20 % vs 27 %	8.7 vs 6.9 vs 5.9	19.2 vs 17.9 vs 17.2	18 % vs 10 % vs 21 %	33
Ducreux M	3	XELOX vs FOLFOX6	France	306	0–2	32–84 (65)	42 % vs 46 %	8.9 vs 9.3	20.1 vs 18.9	11.0 % vs 25.5 %	15
BEV											
AVF2107g Hurwitz H	3	IFL vs IFL + Bev	United States, Australia, New Zealand	813	0–1	18–(59)	35 % vs 45 %	6.2 vs 10.6	15.6 vs 20.3	-	17
E3200 Giantonio BJ	3	FOLFOX4 vs FOLFOX4 + BEV vs BEV	United States, South Africa	829	0–2	21–85 (61)	8.6 % vs 22.7 % vs 3.3 %	4.7 vs 7.3 vs 2.7	-	9.2 % vs 16.3 % vs 0.8 %	18
FIRE-3 Heinemann V	3	FOLFIRI + cetuximab vs FOLFIRI + BEV	Germany, Austria	592	0–2	27–79 (65)	62 % vs 58 %	10.0 vs 10.3	28.7 vs 25.0	0.7 % vs 1.4 %	4
CALGB/SWOG 80405^b^	3	FOLFIRI or mFOLFOX6 + cetuximab vs FOLFIRI or mFOLFOX6 + BEV	United States	1137	0–1	20–89 (59)	-	10.5 vs 10.8	29.9 vs 29.0	12 % vs 14 %	5
XELOX + BEV											
Wong NS	2	XELOX + BEV	United States	50	0–2	24–81 (55)	50 %	10.3	23.3	14 %	11
TREE-2 Hochster HS	2	mFOLFOX6 + BEV vs FOL + BEV vs XELOX + BEV	United States	223	0–1	30–85 (61)	52 % vs 39 % vs 46 %	9.9 vs 8.3 vs 10.3	26.1 vs 20.4 vs 24.6	11 % vs 9 % vs 11 %	33
16966 trial Saltz LB	3	FOLFOX4/XELOX vs FOLFOX4/XELOX + BEV	Worldwide	1401	0–1	18–86 (60)	38 % vs 38 %	8.0 vs 9.4	19.9 vs 21.3	-	27
Elderly											
ASCA trial Munemoto Y	2	XELOX + BEV	Japan	36	0.–1	75–86 (78)	56 %	11.7	22.9	13.9 %	-
SGOSG-CR0501 Matsumoto T	2	UFT + LV	Japan	21	0–2	75–83 (79)	33 %	5.3	18	0 %	21
Feliu J	2	Capecitabine + BEV	Spain	59	0–2	73–79 (75)	34 %	10.8	18.0	0 %	24
Feliu J	2	XELOX	Spain	54	0–2	70–82 (76)	36 %	5.8	13.2	2 %	25
BECOX Feliu J	2	XELOX + BEV	Spain	69	0–1	70–85 (75)	31 %	11.1	20.4	4 %	28
BASIC trial Yoshida M	2	S-1 + BEV	Japan	56	0–1	66–85 (75)	57 %	9.9	25.0	0 %	26
AVEX Cunningham D	3	Capecitabine vs capecitabine + BEV	Worldwide	280	0–2	70–87 (76)	10 % vs 19 %	5.1 vs 9.1	16.8 vs 20.7	0 %	22
FOCUS2 Seymour MT	3	FL vs OxFU vs Capecitabine vs XELOX	United Kingdom	459	0–2	35–87 (74)	11 % vs 38 % vs 14 % vs 32 %	3.5 vs 5.8 vs 5.2 vs 5.8	10.1 vs 10.7 vs 11.0 vs 12.4	0 % vs 1 % vs 0 % vs 4 %	23

*ECOG* the Eastern Cooperative Oncology Group, *PS* performance status, *RR* response rate, *PFS* progression free survival, *OS* overall survival

^a^In the eligibility criteria

^b^Data from the 10th interim analysis (2014)

The overall frequency of grade 3/4 AEs, including hematologic and nonhematologic events, is generally consistent with those of the TREE 1/2 trials, an earlier phase I/II trial conducted in Japan and the BECOX study conducted in Spain [28, 33, 34]. The most characteristic finding here was that the incidence of grade-1 neuropathy reached 83.3 %, and that of grades-3/4 neuropathy was 13.9 %. Cumulative neuropathy represents one of the major problems related to long-term therapy using oxaliplatin-containing regimens for patients with mCRC, which is the main driver for trying to limit the dose of oxaliplatin [12, 14, 28]. The frequency (13.9 %) of G3/4 neuropathy encountered here was higher compared with those reported by earlier studies of Western cohorts (Table 4), although dose reduction and discontinuation of oxaliplatin was strictly defined in the study protocol [13, 28, 35]. A pilot study evaluating the safety of XELOX plus bevacizumab conducted in Japan reported a 17 % frequency of neuropathy G3/4 17 %, indicating that the frequency of severe neuropathy induced by XELOX plus bevacizumab differs between Western and Japanese patients [34]. Haller et al. showed the regional differences in tolerability of XELOX between the United States, East Asia, and the rest of the world [36]. Japanese patients experienced fewer G3/4 AEs during XELOX treatment compared with those from other regions, but no detailed data for neuropathy was provided.

Further, there remains room for discussion about the survival benefit of adding oxaliplatin. For example, in the AVEX study that evaluated capecitabine plus bevacizumab versus capecitabine alone in patients with mCRC aged ≥70 years, the OS of those treated with capecitabine plus bevacizumab is similar OS to that our present study (20.7 months) [22]. Further, the FOCUS2 trial that compared capecitabine plus oxaliplatin with capecitabine alone, found no significant benefit of adding oxaliplatin [23]. Considering the high prevalence of neuropathy here, the benefit of adding oxaliplatin to capecitabine combined with bevacizumab for older Japanese patients with mCRC should be evaluated in clinical trials involving a large number of patients.

During the present study, one patient died because of treatment-related intracerebral bleeding. Although most AEs associated with bevacizumab (hypertension, proteinuria, and bleeding) are manageable, they infrequently lead to death. The patient had normal blood-clotting function as defined by the eligibility criteria, and the onset of intracerebral bleeding occurred after seven cycles of the protocol dose of XELOX plus bevacizumab. However, the overall safety profile of XELOX combined with bevacizumab for patients aged ≥75 years was similar to those of previous clinical trials [27, 33]. From our experience, we propose to monitor neurological signs on each visit and perform cerebral imaging on low threshold in symptomatic patients. In the present study, the incidence of AEs was independent of patients’ sex, age, and BMI. In contrast, low baseline CCr (<64 ml/min) was associated with the frequency of severe AEs, suggesting that baseline CCr should be considered as a determinant of the suitability of treating older patients with XELOX plus bevacizumab. However, further studies of a larger cohort are required.

Our trial achieved response and disease control rates of 55.6 and 91.7 %, respectively. The primary endpoint, median PFS, was 11.7 months (95 % CI 8.0–13.4 months), and the median OS was 22.9 months (95 % CI 17.6–33.0 months). The median PFS in the TREE 2 trial, the earlier Japanese phase I/II trial, and the BECOX study were 10.3, 11.0 and 11.1 months, respectively [21, 28, 33]. The median OS of patients was 22.9 months in our present study, which is somewhat shorter compared with large studies of younger populations. For example, an OS of approximately 29 months was reported by the FIRE-3 and CALGB/SWOG 80405 trials [4, 5]. In contrast, an earlier study of XELOX combined with bevacizumab for Western patients with mCRC aged 75 years demonstrated that OS was 20.4 months [28].

Folprecht et al. analyzed the differences in efficacy of 5-FU-based chemotherapy between age groups >70 years and <70 years with mCRC, and concluded that elderly patients benefit at least to the same extent from palliative chemotherapy with 5-FU compared with younger patients [37]. Recently, Lieu et al. analyzed the large database of the ARCAD Clinical Trials Program and evaluated primary age effects and interactions with sex and PS [38]. They demonstrated that greater age was associated with poorer OS and PFS among treated patients with mCRC independent of sex and PS [38]. The main reason for the survival differences between our study and those of the FIRE-3 and CALGB/SWOG 80405 trials might be accounted for by the age of the patients rather than regional differences, and our results can be considered to reveal a reasonable outcome for patients aged ≥75 years [4, 5].

The present study included some limitations as follows. The relatively small sample size precluded subgroup analysis of age, second-line treatment, and renal function. We selected patients according to strict eligibility criteria to ensure consistency with those of younger individuals. Therefore, these criteria may not be applicable to routine clinical practice. In addition, serial data were unavailable for blood cholesterol, triglyceride, and glucose concentrations that are influenced by capecitabine. The discussion might be limited due to lack of data on RAS/BRAF status. No elderly specific evaluation was conducted though the comprehensive geriatric assessment would have been of high value to learn about factors that are specific to the older patient population which could affect treatment outcome. Because the study protocol had no provisions regarding the second-line treatment, the detailed information of second-line treatment is unavailable. We were unable to determine the survival benefit of XELOX plus bevacizumab because this was a single-arm study.

## Conclusions

Our results indicate that XELOX combined with bevacizumab was well tolerated by selected Japanese patients aged ≥75 years with mCRC. Therefore, XELOX plus bevacizumab should not be withheld from these patients because of age alone. The survival benefit of this regimen must be determined by further controlled clinical trials.

## Additional file

Additional file 1: Figure S1. Receiver operating characteristic curve and waterfall plot. (a) Receiver operating characteristic curve for baseline CCr as a predictor of AEs ≥ grade 3. The AUC and optimal cutoff values were 0.69 and 64 ml/min, respectively. (b) Waterfall plot of maximum percentage tumor shrinkage. Progressive disease was not detected, and lesions with shrinkage of ≥30 % were present in 20 patients (55.6 %). (TIFF 6032 kb)

## Abbreviations

mCRC: Metastatic colorectal cancer
5-FU: 5-fluorouracil
OS: Overall survival
XELOX: Capecitabine and oxaliplatin
FOLFOX: Fluorouracil, folinic acid and oxaliplatin
PFS: Progression-free survival
UMIN: University Hospital Medical Information Network
ECOG: Eastern Cooperative Oncology Group
PS: Performance status
RECIST: Response evaluation criteria in solid tumors
CCr: Creatinine clearance
AE: Adverse effect
CT: Computed tomography
ASA: American Society of Anesthesiologist
BMI: Body mass index
TTF: Time to treatment failure
CI: Confidence interval
AUC: Area under the curve
CR: Complete response
PR: Partial response
SD: Stable disease
