# Supplementary figures and images for: Capecitabine and oxaliplatin combined with bevacizumab are feasible for treating selected Japanese patients at least 75 years of age with metastatic colorectal cancer
Source: BMC Cancer. 2015 Oct 24;15:786. doi: 10.1186/s12885-015-1712-0 (PMC4619505; doi:10.1186/s12885-015-1712-0)

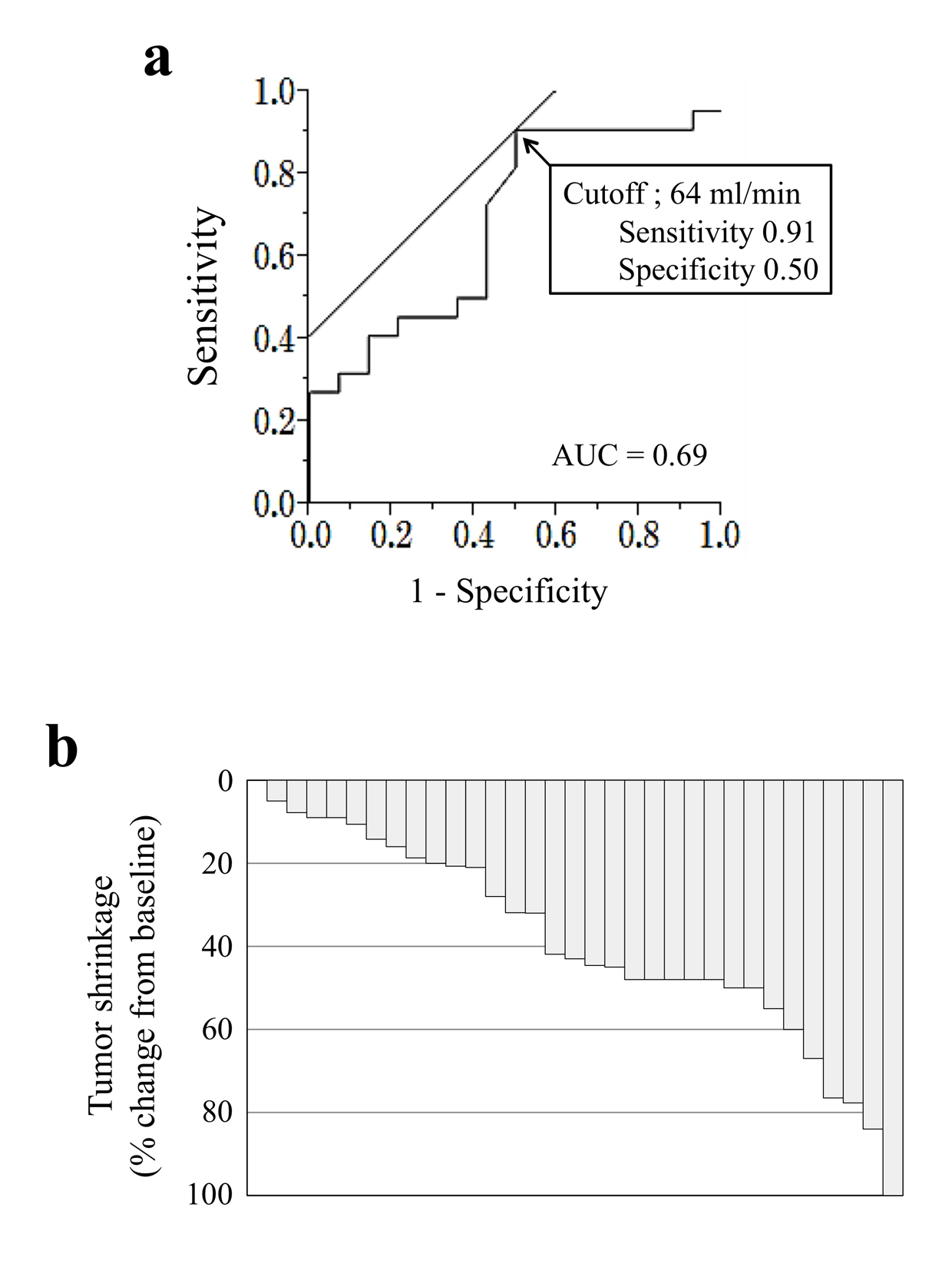

Supplement: Additional file 1: Figure S1. — Receiver operating characteristic curve and waterfall plot. (a) Receiver operating characteristic curve for baseline CCr as a predictor of AEs ≥ grade 3. The AUC and optimal cutoff values were 0.69 and 64 ml/min, respectively. (b) Waterfall plot of maximum percentage tumor shrinkage. Progressive disease was not detected, and lesions with shrinkage of ≥30 % were present in 20 patients (55.6 %). (TIFF 6032 kb) [file 12885_2015_1712_MOESM1_ESM.tiff]
